# Evaluation of Lure and Dispenser Combinations for *Halyomorpha halys* (Hemiptera: Pentatomidae) Trapping

**DOI:** 10.3390/insects16040341

**Published:** 2025-03-25

**Authors:** Vito Antonio Giannuzzi, Valeria Rossi, Rihem Moujahed, Adriana Poccia, Florinda D’Archivio, Tiziano Rossi Magi, Elena Chierici, Luca Casoli, Gabriele Rondoni, Eric Conti

**Affiliations:** 1 Department of Agricultural, Food and Environmental Sciences, University of Perugia, Borgo XX Giugno, 74, 06121 Perugia, Italy; vitoantonio.giannuzzi@dottorandi.unipg.it (V.A.G.); valeria.rossi@unipg.it (V.R.); adriana.poccia@dottorandi.unipg.it (A.P.); florinda.darchivio@gmail.com (F.D.); tizianorossimagi97@gmail.com (T.R.M.); elenachierici9@gmail.com (E.C.); eric.conti@unipg.it (E.C.); 2 Russell IPM Ltd., Deeside CH5 2NU, UK; rihem@russellipm.com; 3 Consorzio Fitosanitario di Reggio Emilia, 42124 Reggio Emilia, Italy; luca.casoli@regione.emilia-romagna.it

**Keywords:** aggregation pheromone, biodegradable releaser, brown marmorated stink bug, IPM, stink bugs

## Abstract

The brown marmorated stink bug (BMSB), *Halyomorpha halys* (Stål) (Hemiptera: Pentatomidae), is a polyphagous pest native to East Asia and invasive in many countries. Various approaches have been used to control its spread, including IPM strategies. Monitoring pest populations is the starting point for developing an appropriate control strategy. The most common attractant for *H. halys* is based on its two-component aggregation pheromone ((3S,6S,7R,10S)-10,11-epoxy-1-bisabolen-3-ol and (3R,6S,7R,10S)-10,11-epoxy-1-bisabolen-3-ol), in combination with the synergist methyl (2E,4E,6Z)-2,4,6-decatrienoate. The aim of this research was to evaluate the most effective combination of the pheromone and the synergist for different types of adjuvants and dispensers, i.e., Blister Pack, Wax Tablet, and Polymers (Non-Biodegradable and Biodegradable Polymers). Field trials were carried out in Northern Italy, during summer and autumn 2023 and 2024. The effectiveness of the different combinations was evaluated on the basis of the number of *H. halys* individuals caught in sticky traps baited with the different treatments, separated by 50 m each. Individuals actively collected in the surrounding vegetation were also monitored. During the first year, the Non-Biodegradable Polymer performed better than the others. In the second year, *H. halys* captures were quite similar among the tested dispensers, and all were higher than the control. Captures of non-target stink bugs and other bugs of the Coreidae family were evaluated but, in most cases, were negligible. Under laboratory conditions, the release rate of the chemical compounds differed depending on the type of dispenser. Our results confirm the specificity of the semiochemical lure combination tested towards *H. halys* and its persistence in the field for at least 12 weeks. The results could be useful for improving the effectiveness of *H. halys* trapping, as well as for attract-and-kill or push–pull strategies of the pest.

## Simple Summary

The use of traps in pest monitoring represents a fundamental aspect of IPM. For several herbivorous insects, the identification of the optimal combination of a chemical attractant with a dispenser is crucial. Here, we evaluated the efficacy of different combinations of semiochemical mixtures (a pheromone and a synergist) and dispensers, through field experiments and laboratory assays, with the aim of improving the attraction of the invasive brown marmorated stink bug, *Halyomorpha halys* (Hemiptera: Pentatomidae). Our findings may be useful to improve *H. halys* trapping, pest monitoring, and the development of control programs based on attract-and-kill or push–pull strategies within IPM.

## 1. Introduction

The increase in the spread of invasive species due to globalization and climate change has been a major threat to the world’s economy and biodiversity [1]. Alien species harm biodiversity by competing with native species and altering agroecosystems, eventually leading to economic losses [2]. To contain their spread in agroecosystems, the development of suitable IPM strategies is needed [3]. Early monitoring provides valuable insights on pest populations, enabling more efficient application of control measures, such as optimizing the timing and frequency of pesticide application [4,5]. 

However, monitoring invasive pests can be challenging due to limited knowledge of their behavior and ecology in their introduction habitats [6]. Semiochemical-based tactics have recently gained importance in IPM, as pheromones and allelochemicals are commonly used for monitoring and control of many insect species [7]. Unlike pesticides, pheromones act on behavior, are effective at very low doses, and less prone to causing the development of insect resistance [8]. One of the primary goals of pheromone research is the identification of species-specific attractants, so to prevent trapping of non-target species that potentially exert important functional roles [9]. Due to the limited persistence of pheromones in the field, their application must be carefully planned. Trap efficiency is influenced by factors, such as pheromone dispenser type and trap positioning [10], as well as environmental conditions, such as relative humidity and temperature [11]. Therefore, it is essential to use dispensers that continuously release a small amount of pheromone over a prolonged time period [12].

The goal of this study was to evaluate, under field and laboratory conditions, the effectiveness and specificity of different pheromonal component ratios and new dispensers in attracting the invasive brown marmorated stink bug, *Halyomorpha halys* (Stål) (Hemiptera: Pentatomidae). We also investigated the possible attraction of native stink bugs (Pentatomoidea superfamily) and leaf-footed bugs (Coreidae family) as non-target species. *Halyomorpha halys* is native to East Asia and has spread to many countries in Europe and North America, where it is considered one of the most important threats to agriculture [13,14,15,16,17]. Its extensive polyphagous diet results in the infestation of over 300 wild and cultivated host plants, including economically important tree fruits, vegetables, field crops, and ornamentals [18,19]. Since its arrival in Italy in 2012, *H. halys* has caused significant threats to agricultural production, particularly in fruit orchards in the northern regions, with an estimated economic loss of EUR 590 million in 2019 [20]. 

The monitoring of *H. halys* is typically carried out using pyramid or sticky traps [21,22,23,24,25] baited with the two-component aggregation pheromone (3S,6S,7R,10S)-10,11-epoxy-1-bisabolen-3-ol and (3R,6S,7R,10S)-10,11-epoxy-1-bisabolen-3-ol (PHER) and, as a synergist, the aggregation pheromone of *Plautia stali* (Scott) (Hemiptera: Pentatomidae), methyl (2E,4E,6Z)-2,4,6-decatrienoate (MDT) [21,26,27]. Clear sticky traps still represent the best choice for farmers and are considered to be more practical than black pyramid traps [22,25,28]. Bi-modal and multimodal traps, combining different stimuli, have also been evaluated [29,30,31]. In previous research, an additional olfactory stimulus, namely ethyl (2E,4E,6Z)-decatrienoate (EDT), showed a potential for application in attractant blends [6], but its inclusion in baits did not significantly increase trap captures compared to the combination of PHER and MDT [32].

The evaluation of the best combination of attractants and dispensers (all produced by Russel IPM) was carried out in a two-year field trial in summer–autumn 2023 and 2024, in a protected natural reserve area located in Northern Italy. Our results provide further confirmation of the reliability of the *H. halys* aggregation lure and can be used as a basis for future push–pull or attract-and-kill strategies to control this species.

## 2. Materials and Methods

### 2.1. Field Location

This study was conducted over 2 years (2023, 2024) in a natural area (approximatively 100 ha) located between Campogalliano (Modena) and Rubiera (Reggio Emilia), in Northern Italy. This location is part of the Curiel Lakes area (coordinates of the central area: 44°39′57.6″ N, 10°49′12.0″ E) and is characterized by stable populations of *H. halys* over recent years. The landscape is characterized by an intense agricultural activity, but the area under consideration is dominated by wild plant species. The vegetation consists of mixed shrubs and trees, including *Acer* spp., *Aesculus hippocastanum* L., *Ailanthus altissima* (Mill.) Swingle, *Amorpha fruticosa* L., *Carpinus* spp., *Celtis australis* L. subsp. *australis*, *Cornus* spp., *Corylus avellana* L., *Cotinus coggygria* Scop., *Crataegus monogyna* Jacq., *Euonymus europaeus* L., *Fraxinus* spp., *Juglans* spp., *Malus domestica* (Suckow) Borkh., *Morus nigra* L., *Phragmites australis* (Cav.) Trin. ex Steud., *Populus* spp., wild *Prunus* spp., *Pyracantha* spp., wild *Pyrus* spp., *Quercus* spp., *Rhamnus cathartica* L., *Robinia* spp., *Rosa canina* L., *Rubus ulmifolius* Schott, *Tilia* spp., and *Ulmus* spp. All these species are known hosts of *H. halys* [33,34]. In this area, *H. halys* traps were placed along a pedestrian walkway, as described in Section 2.2.

### 2.2. Field Experiments

In 2023, the experiment was carried out from August to November (12 weeks in total). Different amounts of PHER and MDT were loaded onto three types of dispensers: a Blister Pack (BLS), Wax Tablet (WXT), and Non-Biodegradable Polymer (NBP) (Table 1 and Figure S6). For each dispenser, PHER and MDT were loaded in two separate elements (“Dual Lure”). Specifically, the BLS was composed of two plastic shells (each of 45 mm diam., 10 mm thick), each with a semipermeable side allowing the release of a compound (PHER or MDT) from a cardboard disk (each of 40 mm diam., 3 mm thick) placed inside. In the WXT, the two lures PHER and MDT were loaded onto separate wax matrixes (20 mm diam., 20 mm thick) and placed inside satin bags (70 mm length × 70 mm width). NBP comprised two polymer strips containing PHER (100 mm length × 30 mm width × 3 mm thick) and MDT (150 mm length × 30 mm width × 3 mm thick), respectively. A Fatty acid Methyl ester (FM) was included in the NBP matrix as an adjuvant. Different ratios of PHER/MDT lure were formulated in four combinations: 10/125 mg (formulation 1), 15/225 mg (formulation 2), 20/200 mg (formulation 3), and 20/300 mg (formulation 4). 

In 2024, the experiment was carried out from July to October, for 14 weeks in total. Only one combination of PHER/MDT was used (10/125 mg), with the addition of different adjuvants (Table 2). The Blister Pack was produced similarly to the previous year, but with a 6 mm cardboard disk. For polymer production, a bio-polymer (BIP) was used, which was made from a biodegradable and compostable fossil-based plastic strip. This represents an environmentally sustainable evolution of the NBP. Moreover, PHER and MDT were loaded in each dispenser together (Single Lure, “SL”) or separately (Dual Lure, “DL”) (Figure S7). BIP_SL (200 mm length × 30 mm width) had a thickness of 7 mm, while BIP_DL (PHER: 100 mm length × 30 mm width; MDT: 200 mm length × 30 mm width) had a thickness of 4 mm for PHER and 7 mm for MDT. In the BLS, Mineral Oil (MO), a Fatty acid Isopropyl ester (FI), and a Fatty acid Methyl ester (FM) were added as adjuvants. In BIP, FM or FM with Lignocellulosic Flour (ML) was added (Table 2). An additional treatment (BIP_DL_MLM) was constituted by PHER without adjuvants and MDT with FM + Lignocellulosic Flour adjuvants.

In 2023 and 2024, lures were used in association with clear sticky traps (24.5 × 40 cm, Halys Trap, Serbios Srl, Badia Polesine (RO), Italy), where two panels were assembled to make both sides sticky. The traps were hung horizontally at 1.5–2 m above the ground inside the canopy of trees or bushes. Unbaited traps served as a control in the trial. Traps were deployed along the pedestrian walkway, spaced 50 m apart and checked weekly. For each treatment, three replications were conducted in a randomized block design. Every three weeks, sticky panels were replaced and treatments within each block were re-randomized.

Field monitoring was conducted weekly by checking the traps and actively inspecting the surrounding area, as the aggregation pheromone attracts individuals close to the release source [35]. Adults and nymphs of *H. halys* and of other non-target stink and leaf-footed bug species captured on the clear sticky traps were counted and removed. An entomological umbrella (50 × 50 cm) was used to conduct a 5 min survey within a 10 m radius of the trap, recording adults and nymphs of *H. halys* and other non-target species.

In order to assess possible fluctuations in *H. halys* populations between the two years, monitoring data from the CIMICE.NET project (data available at https://big.csr.unibo.it/projects/cimice/monitoring.php, accessed on 5 November 2024) [24] were considered. Given their proximity to the monitoring area, traps MO37, MO02, MO17, and MO28 from the CIMICE.NET project were used as a reference to analyze the BMSB population in 2023 and 2024.

### 2.3. Laboratory Evaluation of Attractant Residual Quantity

Fresh dispensers, similar to those tested in the field experiments, were analyzed to assess the release rate of PHER and MDT in laboratory conditions. 

In 2023, two replicates of each treatment were placed in an incubator with a constant air flow at 30 °C and a light–dark photoperiod of 12 h:12 h. Every week for 12 weeks, two samples of each treatment were collected and prepared for volatile extraction and subsequent analysis. After the extraction, the samples were discarded. The preparation for volatile collection followed different protocols according to the different type of dispenser being tested. For the BLS, the 3 mm cardboard disks were cut into 8 pieces, placed in a vial with 10 mL of hexane and extracted for 24 h. For PHER, 1 mL of the solution was transferred to another vial before adding 1 mL of internal standard solution (1 mg of lauryl acetate/mL) and 8 mL of hexane, for a total volume of 10 mL. For MDT, 0.8 mL of the initial solution was transferred to another vial with 1 mL internal standard solution and 8.2 mL of hexane, for a total volume of 10 mL. In both cases, after the dilution, 1 mL was transferred into an autosampler vial for analysis. Regarding WXT, absolute ethanol was used as the solvent. A single tablet was placed in a vial with 10 mL of ethanol, sonicated for 15 min, and extracted for 24 h. Then, 1 mL (for PHER) or 0.8 mL (for MDT) was transferred into a new vial with 1 mL of internal standard solution and 8 mL (PHER) or 8.2 mL (MDT) of hexane. After that, 1 mL of each solution was transferred into an autosampler vial for analysis. In terms of the NBP, the sample was cut into small pieces. For PHER, 1 g was placed into a vial with 1 mL of internal standard solution and 9 mL hexane, for a total volume of 10 mL. For MDT, 1 g of the cut polymer was placed in a vial with 10 mL of hexane and extracted for 24 h. After extraction, 2.8 mL of the solution was transferred into a new vial with 1 mL of internal standard solution and 6.2 mL of hexane (total volume of 10 mL). After that, 1 mL of each solution was transferred into an autosampler vial for analysis. 

In 2024, three replicates of each treatment were placed inside an incubator with a constant air flow at 27 °C and a light–dark photoperiod of 12 h:12 h. Every week for 12 (BIP) or 12 to 15 weeks (BLS), three samples of each treatment were collected and prepared for volatile extraction and further analysis. After the extraction, the samples were discarded. Preparation for volatile extraction followed different protocols depending on the type of dispenser being studied. For the BLS, the 6 mm cardboard disk was cut and placed into a vial before adding 1 mL of internal standard solution (1 mg of lauryl acetate/mL) and 14 mL of hexane. Regarding BIP (10 g of weight), the whole strip was cut into 2–3 mm cubes and placed into two vials which thus contained 5 g each, to which 1 mL of internal standard solution and 9 mL of hexane were added. The vials were left to extract for 12 h and then 1 mL of solution was transferred into an autosampler vial for analysis. The single Lure with ML (BIP_SL_ML) was not evaluated due to the lack of available samples. In addition, the Dual Lure with ML added only on the MDT (BIP_DL_MLM) was not evaluated since the decrease rate of the two components was already assessed within the plain Dual Lure (BIP_DL_00), for PHER, and the Dual Lure with ML (BIP_DL_ML), for MDT.

The same procedure was used to extract the remaining compounds from the lures used for the 2024 field trial at the end of the 14 weeks. Lures were collected from the field and immediately stored at low temperature (−20 °C) and then analyzed for volatile extraction.

All the extracts were analyzed using gas chromatography (7820A GC, Agilent Technologies, Wilmington, DE, USA) on a Zebron ZB-WAXplus GC column (30 m × 0.32 mm × 0.25 μm film thickness) and the amount of PHER and MDT released from each lure was quantified. In total, 1 μL of the sample was injected into the column, which was programmed for 20.75 min with the following temperature specifications: an initial temperature of 80 °C, an increase of 10 °C/min to 150 °C, an increase of 8 °C/min to 220 °C, and a held temperature of 220 °C for 5 min. The injector temperature was 250 °C, the column flow 2 mL/min, and the purge flow 40 mL/min.

The release rates of PHER and MDT, as well as the residual pheromone load in the dispensers over time, were determined using calibration curves specific to each compound. For each MDT and PHER, two calibration curves were constructed to cover the entire range of expected molecule concentrations. For MDT, the first curve was fitted using 0.1, 1, 10, 20, 30, 40, and 50 mg of the compound, while the second curve included 50, 100, 150, 200, 250, and 300 mg of the same compound. For PHER, the first curve was fitted with 0.1, 1, 2.5, 5, 7.5, and 10 mg, whereas the second curve used 10, 12.5, 15, 17.5, and 20 mg of the compound. The calibration curve method was also used to quantify residual pheromone amounts in the dispensers used in the field in 2024. Separate calibration curves were generated for MDT and PHER at the following doses: 0.1, 0.5, 1, 1.5, 2, 2.5, and 5 mg (MDT), and 0.1, 0.5, 1, 1.5, 2, 2.5, and 3 mg (PHER). The internal standard was added to each solution to improve the accuracy of the quantification. Sample concentrations were calculated using the appropriate calibration equations derived from the curves. Specifically, the analyte concentration in each sample was calculated based on its corresponding peak area using the following equation:

*Peak area* = *aC* + *b* (1)

where *a* is the slope of the calibration curve, *C* is the concentration of the compound, and *b* is the intercept of the calibration curve.

### 2.4. Statistical Analysis

Field data were analyzed using linear models to assess the number (log + 1 transformed) of *H. halys* adults and juveniles, and other stink and leaf-footed bugs captured in the sticky traps or present in the surrounding vegetation (Tables S10–S13). For 2023, trap captures and abundance in the surrounding vegetation were evaluated to determine the influence of the PHER/MDT ratios and the type of dispenser used. For 2024, captures and abundance were evaluated to assess the influence of the number of elements in each dispenser and the type of adjuvants.

In addition, Spearman correlation analysis was performed to investigate a possible correlation between trap captures and the density of *H. halys* in the surrounding vegetation, both for adults and juveniles. The results are presented in the Supplementary Materials (Tables S14 and S15). For the laboratory trials, linear models were fitted to test the release rate of PHER or MDT over time. Data were log + 1 transformed prior to analysis. Moreover, for field and laboratory data, differences between treatments were evaluated by a multiple comparisons procedure. Analyses were performed using the R statistical environment and packages “emmeans” and “multcomp” [36,37,38].

## 3. Results

### 3.1. Field Trials

The 2023 monitoring campaign showed significant differences in *H. halys* captures (only in adults) among treatments (Table 3). In particular, NBP baits loaded with PHER/MDT ratios of 20/300 mg (NBP_4_FM) and 20/200 mg (NBP_3_FM) led to the greatest attraction to the traps compared to other treatments and the control. Conversely, WXT in all formulations (i.e., WXT_1_00, WXT_2_00, WXT_3_00, and WXT_4_00) and BLS with a PHER/MDT ratio of 15/225 mg (BLS_2_00) exhibited comparatively inferior performances, similar to the control. The other treatments showed an intermediate attractiveness. Regarding the presence of adults in the surrounding vegetation, only NBP_4_FM showed greater *H. halys* (adults) attraction compared to the control (Table 3). In terms of the non-target species, only a few specimens were caught in the traps and variations between treatments were not registered (Table S1). 

The 2024 monitoring campaign also showed differences in adult trapping (Table 4). BIP baits were all clearly different from the control except BIP_DL_00. The BLS baits BLS_SL_FM, BLS_SL_MO, BLS_SL_FI, and BLS_DL_00 did not behave differently from the control, with only the remaining treatments showing better attraction. Concerning the abundance of *H. halys* adults in the surrounding vegetation, only BLS_DL_MO performed better than the control. In terms of juvenile abundance, no differences were observed among treatments. Only a few individuals of non-target species were caught in the traps (Table S2). For several treatments, trap captures and stink bug abundance in the surrounding vegetation were positively correlated (Tables S14 and S15). 

Overall, a decrease in captures and abundance of *H. halys* individuals was detected between 2023 and 2024. This decrease was not limited to the fields under study, but affected a wider area, as underscored by the trapping data from the monitoring network [24]. According to the available data, the average reduction in insect captures was ca. 16%.

### 3.2. Laboratory Residual Quantity of PHER and MDT

Laboratory analysis of the release rate revealed a decrease in the remaining percentage of volatile compounds over time for all dispensers (results of statistical analyses in Tables S3–S7). In 2023, the release rate of PHER in BLS and NBP, and of MDT in BLS, did not differ among treatments (Tables S3 and S5). Concerning WXT, release rate was higher for PHER in WXT_4_00 and MDT in WXT_2_00 compared to the other treatments (Table S4). As for NBP, the release rate of MDT was higher in NBP_3_FM (Table S5). 

In 2024, the PHER and MDT release of BLS and BIP showed significant differences between treatments (Tables S6 and S7). In BLS, the release of PHER was higher in the Dual Lure with MO (BLS_DL_MO) and the release of MDT was higher in the Single Lure (BLS_SL_00) compared to the other treatments (Table S6). Concerning BIP, the release of PHER was higher in the Dual Lure with ML (BIP_DL_ML) and the release of MDT was higher in the Dual Lure with ML (BIP_DL_ML), Dual Lure with FM (BIP_DL_FM), plain Single Lure (BIP_SL_00), and Single Lure with FM (BIP_SL_FM) (Table S7). 

In 2024, the release rate of the BLS dispensers was higher in terms of PHER in the Dual Lure with FM (BLS_DL_FM) and the Dual Lure with FI (BLS_DL_FI), while it was higher for MDT in the plain Dual Lure (BLS_DL_00), plain Single Lure (BLS_SL_00), and Single Lure with MO (BLS_SL_MO) compared to the other treatments (Table S8). In BIP, the release rate was higher for PHER in the Single Lure with FM (BIP_SL_FM) and the plain Dual Lure (BIP_DL_00), while the release rate was higher for MDT in the Dual Lure with ML (BIP_DL_ML) compared to the other treatments (Table S9). 

## 4. Discussion

The 2023 field trial demonstrated variations in trap captures between treatments. The use of different ratios of PHER and MDT did not result in statistically different attractions of *H. halys*, and this is in accordance with prior studies [32]. In contrast, the dispenser type had a significant effect on insect attraction. NBP demonstrated good performance in all four formulations, with notable efficacy observed at doses of PHER/MDT 20/300 mg (NBP_4_FM) and 20/200 mg (NBP_3_FM). However, the field trapping only lasted up to 10 weeks (late October–early November), which is about the time when *H. halys* disappeared for overwintering. Therefore, we cannot rule out the possibility that the lures may have remained attractive for more than 10 weeks. Conversely, WXT exhibited a notable lack of attractiveness across all PHER/MDT ratios, strengthening the fact that modifications of the ratio between PHER and MDT have negligible consequences on the attractive potential of this lure, whereas the dispenser is of pivotal importance. Laboratory data on the PHER and MDT release rate obtained in 2023 revealed that lures loaded on WXT persisted for at least 12 weeks. Thus, their low attractiveness in the field could be due to their melting during the warmest weeks of the experiments. This could have resulted in their semiochemical molecules being exposed to the atmosphere and sunlight, affecting their stability and persistence [39,40]. BLS exhibited intermediate behavior compared to the other two dispensers. The release of MDT in the NBP and of MDT and PHER in the BLS was very high under laboratory conditions, and then dropped below 10% after few weeks. According to previous studies [41,42,43,44], the compound release rate is influenced by temperature, with a progressive acceleration as the temperature rises. Therefore, the difference between the laboratory analysis and the field outcomes can be explained by an excessively high constant laboratory temperature (30 °C), which may have influenced the results by accelerating the lure aging process. Indeed, it is unlikely that an average daily temperature of 30 °C was reached for a prolonged period during the 12-week field trial (from late August to mid-November). This can explain the prolonged attractiveness of the traps under field conditions compared to the laboratory.

The 2023 abundance of *H. halys* adults in the surrounding vegetation revealed differences among treatments, similar to those observed for trap captures. Conversely, no significant differences between treatments were recorded for juveniles. Additionally, juvenile captures and their density in the surrounding vegetation dropped during the 4^th^–5^th^ week (around mid to late September). After this period, only adults belonging to the overwintering generation were found. Hence, due to the late start of the monitoring campaign (mid-August), the captures in 2023 include only first-generation adults, second-generation juveniles, and overwintering adults.

Regarding non-target species, when considered all together, they were not attracted to the *H. halys* lures. However, when considered separately, *Nezara viridula* (Linnaeus) (Hemiptera: Pentatomidae) was found to be significantly attracted to NBP baited with PHER/MDT 10/125 mg (NBP_1_FM). Other studies have demonstrated the response of this species to MDT [45,46].

The 2024 field trial showed a differentiation in adult captures between treatments and the control, mostly due to BIP treatments. Variation in the abundance of adults in the surrounding vegetation was also observed. In particular, BLS_DL_MO showed higher efficacy in terms of adult attraction. However, no significant differences among treatments were observed in juvenile trap captures and their density in the surrounding area. No improvement in *H. halys* captures was noticed with Double Lures compared to Single Lures either. Also, given to the relatively limited population of *H. halys* in 2024, it was challenging to discern whether significant variations in captures were strictly related to the presence of adjuvants. Future experiments are needed to clarify the effective role of adjuvants in increasing the efficacy of dispenser attraction. None of the treatments tested in 2024 were particularly attractive to non-target species, confirming specificity of the formulations tested.

Based on the results of the 2023 laboratory residual rate trials, the 2024 investigation was conducted at a constant temperature of 27 °C. This modification, combined with the addition of several adjuvants, a change in polymer matrix (BIP instead of NBP), and the addition of a thicker cardboard layer inside the BLS (6 mm instead of 3 mm), resulted in a consistent improvement in the sustained release of both PHER and MDT. Indeed, the release of both components lasted for at least 12 weeks. These aspects should be considered when designing the best attractant [40]. For example, the BLS loaded with 10/125 mg of PHER/MDT in 2023 (BLS_1_00) was improved in 2024 (BLS_DL_00) by increasing the thickness of the cardboard disk from 3 mm to 6 mm while maintaining the same amount of PHER, MDT, and adjuvants. The laboratory results showed a clear improvement in the releasing performance, with a transition from 0% at week 6 for PHER and at week 4 for MDT in 2023 to a sustained release duration that exceeded 12 weeks in 2024 (analysis in Table S6).

Overall, our results demonstrate that the choice of the right dispenser for holding and releasing insect pheromones is crucial for the effectiveness of pest control strategies [40]. An effective pheromone control strategy requires dispensers that release the pheromone in a controlled manner over time, thus maximizing its effectiveness. A well-designed dispenser ensures pheromone delivery in a way that optimizes attraction, exposing insects to the pheromone at the right time and place. An improper dispenser, on the other hand, can result in reduced attraction, as shown by our study, and attenuated control efficacy. 

The viability of such strategies must be evaluated on a case-by-case basis, considering factors such as the target species, the specific pheromone being used, the control approach, the dispensing method, and the integration of other pest control strategies [8]. Environmental conditions and other influencing factors must also be taken into account [39]. In our research, given the late monitoring campaign, MDT may have played a key role in attracting insects. The synergistic effect of the aggregation pheromones of *H. halys* and *P. stali* in attracting *H. halys* specimens has been widely confirmed in previous studies [6,17,21]. This combination enables effective capture throughout the entire season, as PHER is more attractive in the early and mid-season, while MDT is more effective in the late season [21]. Heterospecific pheromone attraction is common in stink bugs and it may result in the aggregation of different species, providing mutual benefits such as an improved food supply and overwintering site [47].

Further field trials will be necessary to identify the optimal lure, with an emphasis on reducing the treatment numbers to be tested, so as to eventually improve the statistical power of these studies. In fact, the space constraint in the study area did not permit an increase in replications of the defined number of experimental treatment combinations. Once identified, the best-performing dispenser may be used also in future attract-and-kill strategies. Examples of such promising control measures against *H. halys* are becoming more frequent [48,49,50,51]. In addition, functional dispensers could be used in combination with repellent molecules (e.g., terpenes, alarm pheromones, host plant semiochemicals) in push–pull strategies [52,53]. Such IPM approaches would also likely improve the control of *H. halys* because they are expected to cause little interference in complementary control practices, such as biocontrol [29]. The improvement of pheromone ratios and dispensers also allows for the possibility of attracting different genotypes, as *H. halys* populations have been found to comprise different genetic strains [54,55,56].

## Figures and Tables

**Table 1:** Overview of the different treatments evaluated from August to November 2023. “BLS” stands for Blister Pack, “WXT” for Wax Tablet, and “NBP” for Non-Biodegradable Polymer. In addition to control (CNT, no lure, no dispenser), four different combinations of PHER/MDT were evaluated: 10/125 mg (formulation 1), 15/125 mg (formulation 2), 20/200 mg (formulation 3), and 20/300 mg (formulation 4). The NBP dispenser included a Fatty acid Methyl ester (FM) as adjuvant, while no adjuvants were added to the others (00).

Treatment	Dispenser type	PHER/MDT	Lure	Adjuvant
CNT	Control	-	-	-
BLS_1_00	Blister Pack	10 mg/125 mg	Dual	None
BLS_2_00	Blister Pack	15 mg/225 mg	Dual	None
BLS_3_00	Blister Pack	20 mg/200 mg	Dual	None
BLS_4_00	Blister Pack	20 mg/300 mg	Dual	None
WXT_1_00	Wax tablet	10 mg/125 mg	Dual	None
WXT_2_00	Wax tablet	15 mg/225 mg	Dual	None
WXT_3_00	Wax tablet	20 mg/200 mg	Dual	None
WXT_4_00	Wax tablet	20 mg/300 mg	Dual	None
NBP_1_FM	Non-Biodegradable Polymer	10 mg/125 mg	Dual	Fatty acid Methyl ester
NBP_2_FM	Non-Biodegradable Polymer	15 mg/225 mg	Dual	Fatty acid Methyl ester
NBP_3_FM	Non-Biodegradable Polymer	20 mg/200 mg	Dual	Fatty acid Methyl ester
NBP_4_FM	Non-Biodegradable Polymer	20 mg/300 mg	Dual	Fatty acid Methyl ester

**Table 2:** Overview of the different treatments evaluated from July to October 2024. “BLS” stands for Blister Pack, “BIP” for Bio-Polymer, “SL” for Single Lure, “DL” for Dual Lure, and “CNT” for Control (no lure, no dispenser). Different adjuvants were added together with PHER/MDT (10 mg/125 mg): Mineral Oil (MO), a Fatty acid Isopropyl ester (FI), a Fatty acid Methyl ester (FM), and Fatty acid Methyl ester with Lignocellulosic Flour (ML). When no adjuvants were added, it was referred to as “00”. The Dual Lure BIP with PHER without adjuvants and MDT with ML was denoted as “BIP_DL_MLM”.

Treatment	Dispenser Type	PHER/MDT	Lure	Adjuvant
CNT	Control	-	-	-
BLS_SL_00	Blister Pack	10 mg/125 mg	Single	None
BLS_SL_MO	Blister Pack	10 mg/125 mg	Single	Mineral Oil
BLS_SL_FI	Blister Pack	10 mg/125 mg	Single	Fatty acid Isopropyl ester
BLS_SL_FM	Blister Pack	10 mg/125 mg	Single	Fatty acid Methyl ester
BLS_DL_00	Blister Pack	10 mg/125 mg	Dual	None
BLS_DL_MO	Blister Pack	10 mg/125 mg	Dual	Mineral Oil
BLS_DL_FI	Blister Pack	10 mg/125 mg	Dual	Fatty acid Isopropyl ester
BLS_DL_FM	Blister Pack	10 mg/125 mg	Dual	Fatty acid Methyl ester
BIP_SL_00	Bio-Polymer	10 mg/125 mg	Single	None
BIP_SL_FM	Bio-Polymer	10 mg/125 mg	Single	Fatty acid Methyl ester
BIP_SL_ML	Bio-Polymer	10 mg/125 mg	Single	Fatty acid Methyl ester with Lignocellulosic Flour
BIP_DL_00	Bio-Polymer	10 mg/125 mg	Dual	None
BIP_DL_FM	Bio-Polymer	10 mg/125 mg	Dual	Fatty acid Methyl ester
BIP_DL_ML	Bio-Polymer	10 mg/125 mg	Dual	Fatty acid Methyl ester with Lignocellulosic Flour
BIP_DL_MLM	Bio-Polymer	10 mg/125 mg	Dual	Fatty acid Methyl ester with Lignocellulosic Flour (MDT)

**Table 3:** Trap captures and abundance in the surrounding vegetation (mean ± SE) of *Halyomorpha halys* (adults and juveniles) in 2023. Means within the same column followed by different letters are significantly different according to a linear model followed by a multiple comparisons procedure (significance level α = 0.05). The captures and abundance of juveniles were not significantly different. For treatment details, please refer to Table 1.

	On Trap		Surrounding Vegetation	
Treatment	Adults (Mean ± SE)	Juveniles (Mean ± SE)	Adults (Mean ± SE)	Juveniles (Mean ± SE)
CNT	0 ± 0 f	0.33 ± 0.33	26 ± 4.62 b	23 ± 1.53
BLS_1_00	4.67 ± 0.67 abcd	2.67 ± 0.67	61 ± 7.55 ab	65.67 ± 17.75
BLS_2_00	2.33 ± 1.86 def	2.33 ± 0.88	54.67 ± 19.78 ab	84.33 ± 50.39
BLS_3_00	8 ± 6.51 bcd	5 ± 4.51	57 ± 24.58 ab	66.67 ± 33.89
BLS_4_00	4 ± 2.52 cde	4.33 ± 1.2	50.33 ± 29.34 ab	128 ± 7.21
WXT_1_00	0.33 ± 0.33 ef	0.33 ± 0.33	44 ± 1.53 ab	62.33 ± 31.63
WXT_2_00	2 ± 1 def	2 ± 1.53	54 ± 11.27 ab	33 ± 14.84
WXT_3_00	1 ± 1 def	3.33 ± 1.76	41 ± 27.51 b	79.33 ± 46.09
WXT_4_00	3 ± 1.53 def	2 ± 1.53	75 ± 25.54 ab	86.33 ± 36.44
NBP_1_FM	12.33 ± 1.2 abc	4 ± 3.51	154.67 ± 38.08 ab	108.33 ± 54.96
NBP_2_FM	16 ± 2.65 ab	4.33 ± 3.84	145.33 ± 90.47 ab	91.67 ± 55.32
NBP_3_FM	19.33 ± 2.96 a	5.33 ± 2.6	151.67 ± 49.4 ab	71.67 ± 43.76
NBP_4_FM	19 ± 2.08 a	8.67 ± 4.06	242 ± 71.36 a	150.67 ± 63.83

**Table 4:** Trap captures and abundance in the surrounding vegetation (mean ± SE) of *Halyomorpha halys* (adults and juveniles) in 2024. Means in the same column followed by different letters are significantly different according to a linear model followed by a multiple comparisons procedure (significance level α = 0.05). The captures and abundance of juveniles were not significantly different. For treatment details, please refer to Table 2.

	On Trap		Surrounding Vegetation	
Treatment	Adults (Mean ± SE)	Juveniles (Mean ± SE)	Adults (Mean ± SE)	Juveniles (Mean ± SE)
CNT	0 ± 0 b	0 ± 0	4 ± 2.52 b	12 ± 2.65
BLS_SL_00	5 ± 1 a	8.33 ± 4.48	35 ± 15.5 ab	39.33 ± 20.74
BLS_SL_MO	2.33 ± 1.86 ab	10.33 ± 2.6	29.67 ± 19.7 ab	34.67 ± 9.53
BLS_SL_FI	3 ± 1.73 ab	7.67 ± 3.53	24.33 ± 7.88 ab	58.33 ± 26.52
BLS_SL_FM	1.33 ± 0.88 ab	7 ± 2	25.67 ± 11.86 ab	63.33 ± 33.89
BLS_DL_00	3.33 ± 1.2 ab	6 ± 1.53	37 ± 24.25 ab	38.67 ± 2.85
BLS_DL_MO	8 ± 3.51 a	9 ± 3.46	83.33 ± 47.34 a	58.33 ± 29.16
BLS_DL_FI	5.33 ± 1.45 a	5.33 ± 2.33	16.33 ± 0.67 ab	56 ± 17.21
BLS_DL_FM	4 ± 1.53 a	8.67 ± 3.38	33.67 ± 18.99 ab	32.33 ± 11.67
BIP_SL_00	3.33 ± 0.67 a	4.33 ± 1.67	52 ± 18.88 ab	69 ± 12.34
BIP_SL_FM	3.67 ± 0.88 a	9.67 ± 4.33	13.67 ± 6.17 ab	32 ± 5.57
BIP_SL_ML	4.33 ± 1.2 a	5 ± 4.04	35 ± 9.54 ab	108 ± 54.04
BIP_DL_00	1.67 ± 0.33 ab	6.33 ± 1.33	28.33 ± 13.91 ab	50.67 ± 6.69
BIP_DL_FM	6 ± 2.65 a	9.67 ± 2.96	19.67 ± 0.67 ab	40.67 ± 19.64
BIP_DL_ML	4.67 ± 1.2 a	8 ± 3.61	58.67 ± 41.67 ab	73.33 ± 15.06
BIP_DL_MLM	7.67 ± 2.67 a	8.33 ± 3.84	33.67 ± 11.62 ab	54 ± 11.85

## Data Availability

Data are available upon request to the authors.

## Supplementary Materials

The following supporting information can be downloaded at: https://www.mdpi.com/article/10.3390/insects16040341/s1, Table S1: Trap captures and abundance in the surrounding vegetation (mean ± SE) of native stink and leaf-footed bugs (*Aelia acuminata, Acrosternum heegeri, Arma custos, Dolycoris baccarum, Eurydema ornata, Eysarcoris* spp., *Gonocerus acuteangulatus, Graphosoma italicum, Nezara viridula, Palomena prasina, Peribalus* spp., *Piezodorus lituratus, Raphigaster nebulosa*) in 2023. Means within the same column did not differ according to linear model. For treatment details, please refer to Table 1; Table S2: Trap captures and abundance in the surrounding vegetation (mean ± SE) of native stink and leaf-footed bugs (*Acrosternum heegeri, Arma custos, Coreus marginatus, Eysarcoris* spp., *Gonocerus acuteangulatus, Graphosoma italicum, Nezara viridula, Odontotarsus* spp., *Palomena prasina, Peribalus* spp., *Piezodorus lituratus, Raphigaster nebulosa*) in 2024. Means within the same column did not differ according to linear model. For treatment details, please refer to Table 2; Table S3: Anova table of the linear model investigating the residual quantity of PHER and MDT loaded on BLS for 2023 laboratory trials. Multiple comparisons procedure (significance level α = 0.05) revealed no difference for both PHER and MDT; Table S4: Anova table of the linear model investigating the residual quantity of PHER and MDT loaded on WXT for 2023 laboratory trials. Multiple comparisons procedure revealed differences for PHER (from higher to lower residual percentage: WXT_3_00 (a), WXT_2_00 (a), WXT_1_00 (ab), WXT_4_00 (b)) and MDT (from higher to lower residual percentage and considering the interactions between treatments and week: (WXT_1_00 (a), WXT_4_00 (a), WXT_3_00 (a), WXT_2_00 (b) (significant differences at α = 0.05 were identified by different letters).; Table S5: Anova table of the linear model investigating the residual quantity of PHER and MDT loaded on NBP for 2023 laboratory trials. Multiple comparisons procedure revealed no difference for PHER. Differences were found for MDT: (from higher to lower residual percentage: NBP_1_FM (a), NBP_4_FM (ab), NBP_2_FM (bc), NBP_3_FM (c)) (significant differences at α = 0.05 were identified by different letters).; Table S6: Anova table of the linear model investigating the residual quantity of PHER and MDT loaded on BLS for 2024 laboratory trials. Multiple comparisons procedure revealed differences between treatments for PHER (from higher to lower residual percentage and considering the interactions between treatment and week: BLS_SL_FI (a), BLS_SL_FM (ab), BLS_DL_00 (b), BLS_DL_FI (c), BLS_SL_MO (cd), BLS_DL_FM (d), BLS_SL_00 (d), BLS_DL_MO (e)) and MDT (from higher to lower residual percentage and considering the interactions between treatment and week: BLS_SL_FM (a), BLS_SL_FI (ab), BLS_DL_FM (bc), BLS_DL_FI (c), BLS_SL_MO (d), BLS_DL_MO (d), BLS_DL_00 (e), BLS_SL_00 (f)) (significant differences at α = 0.05 were identified by different letters).; Table S7: Anova table of the linear model investigating the residual quantity of PHER and MDT loaded on BIP for 2024 laboratory trials. BIP_SL_ML was not evaluated due to a deficiency in the experiment, BIP_DL_MLM because the decrease rate of the two components was already evaluated within the treatments BIP_DL_00 (PHER) and BIP_DL_ML (MDT). Multiple comparisons procedure revealed differences between treatments for PHER (from higher to lower residual percentage and considering the interactions between treatment and week: BIP_SL_FM (a), BIP_SL_00 (a), BIP_DL_00 (ab), BIP_DL_FM (bc), BIP_DL_ML (c)) and MDT (from higher to lower residual percentage and considering the interactions between treatment and week: BIP_DL_00 (a), BIP_SL_FM (b), BIP_SL_00 (b), BIP_DL_FM (b), BIP_DL_ML (b)) (significant differences at α = 0.05 were identified by different letters).; Table S8: Anova table of the linear model investigating the residual quantity of PHER and MDT loaded on BLS used in the 2024 field experiment. Multiple comparisons procedure revealed differences between treatments for PHER (from higher to lower residual percentage: BLS_DL_MO (a), BLS_SL_00 (a), BLS_SL_MO (ab), BLS_DL_00 (ab), BLS_SL_FM (bc), BLS_SL_FI (bc), BLS_DL_FM (c), BLS_DL_FI(c) and MDT (from higher to lower residual percentage: BLS_DL_FI (a), BLS_SL_FM (b), BLS_DL_FM (b), BLS_SL_FI (bc), BLS_DL_MO (cd), BLS_DL_00 (d), BLS_SL_00 (d), BLS_SL_MO (d)) (significant differences at α = 0.05 were identified by different letters).; Table S9: Anova table of the linear model investigating the residual quantity of PHER and MDT loaded on BIP used in the 2024 field experiment. Multiple comparisons procedure revealed differences between treatments for PHER (from higher to lower residual percentage: BIP_DL_MLM (a), BIP_DL_ML (b), BIP_SL_00 (bc), BIP_SL_ML (bc), BIP_DL_FM (bc), BIP_SL_FM (c), BIP_DL_00 (c)) and MDT (from higher to lower residual percentage: BIP_SL_00 (a), BIP_DL_MLM (ab), BIP_SL_MWL (bc), BIP_SL_FM (cd), BIP_DL_00 (cd), BIP_DL_FM (cd), BIP_DL_ML (d)) (significant differences at α = 0.05 were identified by different letters).; Table S10: Anova table of the linear model investigating trap captures during 2023 field experiment.; Table S11: Anova table of the linear model investigating abundance in the surrounding vegetation during 2023 field experiment.; Table S12: Anova table of the linear model investigating trap captures during 2024 field experiment.; Table S13: Anova table of the linear model investigating abundance in the surrounding vegetation during 2024 field experiment.; Table S14: Correlation analysis for 2023 trap captures and abundance in the surrounding vegetation.; Table S15: Correlation analysis for 2024 trap captures and abundance in the surrounding vegetation. Figure S1. Residual quantity of MDT and PHER in BLS dispenser during 2023 laboratory trial. Pink = BLS_1_00, black = BLS_2_00, navy-blue = BLS_3_00, gray = BLS_4_00. For details on the compound combinations please refer to Table 1 of the main manuscript. Figure S2. Residual quantity of MDT and PHER in WXT dispenser during 2023 laboratory trial. Pink = WXT_1_00, black = WXT_2_00, navy-blue = WXT_3_00, gray = WXT_4_00. For details on the compound combinations please refer to Table 1 of the main manuscript. Figure S3. Residual quantity of MDT and PHER in EVP dispensers during 2023 laboratory trial. Pink = EVP_1_MP, black = EVP_2_MP, navy-blue = EVP_3_MP, gray = EVP_4_MP. For details on the compound combinations please refer to Table 1 of the main manuscript. Figure S4. Residual quantity of MDT and PHER in BLS dispensers during 2024 laboratory trial. Navy-blue = BLS_SL_00, solid line; BLS_DL_00, dashed line. Black = BLS_DL_MP, solid line; BLS_SL_MO, dashed line. Gray = BLS_SL_IP, solid line; BLS_DL_IP, dashed line. Pink = BLS_DL_MO solid line; BLS_SL_MP, dashed line. For details on the treatments please refer to Table 1 of the main manuscript. Figure S5. Residual quantity of MDT and PHER in BIP dispensers during 2024 laboratory trial. Navy-blue = BIP_DL_MW, solid line; BIP_SL_00, dashed line. Black = BIP_SL_MP. Gray = BIP_DL_00. Pink = BIP_DL_MP. For details on the compound combinations please refer to Table 1 of the main manuscript. Figure S6. Types of dispensers used in the 2023 field trial. From left to right: Blister Pack (BLS), Wax Tablets (WXT), and Non-Biodegradable Polymer (NBP). Figure S7. Types of dispensers used in the 2024 field trial. From left to right: Blister Pack Single Lure (BLS_SL), Blister Pack Dual Lure (BLS_DL), Biodegradable Polymer Single Lure (BIP_SL), and Biodegradable Polymer Dual Lure (BIP_DL).
