# Supplementary material for: Evaluation of Lure and Dispenser Combinations for Halyomorpha halys (Hemiptera: Pentatomidae) Trapping
Source: Insects. 2025 Mar 25;16(4):341. doi: 10.3390/insects16040341 (PMC12028315; doi:10.3390/insects16040341)
Supplement: Supplementary file 1 [file insects-16-00341-s001.zip › insects-3365970-supplementary-03.07.pdf]

Evaluation of Lure and Dispenser Combinations for *Halyomorpha halys* (Hemiptera: Pentatomidae) Trapping

Vito Antonio Giannuzzi [1,†], Valeria Rossi [1,†], Rihem Moujahed [2], Adriana Poccia [1], Florinda D'Archivio [1], Tiziano Rossi Magi [1], Elena Chierici [1], Luca Casoli [3], Gabriele Rondoni [1,*] and Eric Conti [1]

[1] Department of Agricultural, Food and Environmental Sciences, University of Perugia, Borgo XX Giugno, 74, 06121 Perugia, Italy; vitoantonio.giannuzzi@dottorandi.unipg.it (V.A.G.); valeria.rossi@unipg.it (V.R.); adriana.poccia@dottorandi.unipg.it (A.P.); florinda.darchivio@gmail.com (F.D.); tizianorossimagi97@gmail.com (T.R.M.); elenachierici9@gmail.com (E.C.); eric.conti@unipg.it (E.C.)

[2] Russell IPM Ltd., Deeside CH5 2NU, UK; rihem@russellipm.com

[3] Consorzio Fitosanitario di Reggio Emilia, 42124 Reggio Emilia, Italy; luca.casoli@regione.emilia-romagna.it

* Correspondence: gabriele.rondoni@unipg.it

† These authors contributed equally to this work.

Table S1: Trap captures and abundance in the surrounding vegetation (mean ± SE) of native stink and leaf-footed bugs (*Aelia acuminata*, *Acrosternum heegeri*, *Arma custos*, *Dolycoris baccarum*, *Eurydema ornata*, *Eysarcoris* spp., *Gonocerus acuteangulatus*, *Graphosoma italicum*, *Nezara viridula*, *Palomena prasina*, *Peribalus* spp., *Piezodorus lituratus*, *Raphigaster nebulosa*) in 2023. Means within the same column did not differ according to linear model. For treatment details, please refer to Table 1.

	**On Trap**	**Surrounding vegetation**
**Treatment**	**Adults and juveniles (mean ± SE)**	**Adults and juveniles (mean ± SE)**
CNT	0 ± 0	37 ± 8.5
BLS_1_00	0.33 ± 0.33	7.33 ± 2.33
BLS_2_00	1 ± 1	20 ± 3.51
BLS_3_00	0.67 ± 0.67	16.33 ± 6.44
BLS_4_00	2 ± 1.15	16.67 ± 5.36
WXT_1_00	0 ± 0	17 ± 3
WXT_2_00	1 ± 0	27.67 ± 17.64
WXT_3_00	1 ± 1	32.33 ± 4.26
WXT_4_00	0 ± 0	10.67 ± 4.18
NBP_1_FM	1 ± 0	13 ± 3.21
NBP_2_FM	0 ± 0	9.33 ± 4.33
NBP_3_FM	1.33 ± 0.33	9.67 ± 4.63
NBP_4_FM	1.67 ± 1.2	4 ± 0.58

Table S2: Trap captures and abundance in the surrounding vegetation (mean ± SE) of native stink and leaf-footed bugs (*Acrosternum heegeri*, *Arma custos*, *Coreus marginatus*, *Eysarcoris* spp., *Gonocerus acuteangulatus*, *Graphosoma italicum*, *Nezara viridula*, *Odontotarsus* spp., *Palomena prasina*, *Peribalus* spp., *Piezodorus lituratus*, *Raphigaster nebulosa*) in 2024. Means within the same column did not differ according to linear model. For treatment details, please refer to Table 2.

	**On Trap**	**Surrounding vegetation**
**Treatment**	**Adults and juveniles (mean ± SE)**	**Adults and juveniles (mean ± SE)**
CNT	2 ± 2	10 ± 2
BLS_SL_00	1.33 ± 0.33	8.67 ± 3.28

BLS_SL_MO	1.33 ± 0.33	5 ± 1.53
BLS_SL_FI	0 ± 0	9.67 ± 4.67
BLS_SL_FM	1 ± 0.58	7.67 ± 2.03
BLS_DL_00	0 ± 0	5.67 ± 2.03
BLS_DL_MO	1 ± 0	6 ± 3.06
BLS_DL_FI	1.33 ± 0.33	12.67 ± 5.36
BLS_DL_FM	2.33 ± 0.88	11.67 ± 6.77
BIP_SL_00	1 ± 0.58	20.33 ± 9.4
BIP_SL_FM	0.67 ± 0.33	8.33 ± 4.84
BIP_SL_ML	0.33 ± 0.33	8.33 ± 2.03
BIP_DL_00	0.33 ± 0.33	6 ± 0.58
BIP_DL_FM	1.33 ± 0.33	5 ± 2.08
BIP_DL_ML	0.67 ± 0.33	21.33 ± 9.56
BIP_DL_MLM	0.67 ± 0.33	5.33 ± 2.67

Table S3: Anova table of the linear model investigating the residual quantity of PHER and MDT loaded on BLS for 2023 laboratory trials. Multiple comparisons procedure (significance level α = 0.05) revealed no difference for both PHER and MDT.

Compound	Source of variation	Df	Sum Sq	Mean Sq	F value	P value
PHER	Treatment	3	12.38	4.13	2.78	0.046
	Week (log-transformed)	1	196.46	196.46	132.33	< 0.0001
	Residuals	83	123.23	1.49		
MDT	Treatment	3	0.81	0.27	0.60	0.62
	Week (log-transformed)	1	126.32	126.32	279.10	< 0.0001
	Residuals	83	37.57	0.45		

Table S4: Anova table of the linear model investigating the residual quantity of PHER and MDT loaded on WXT for 2023 laboratory trials. Multiple comparisons procedure revealed differences for PHER (from higher to lower residual percentage: WXT_3_00 (a), WXT_2_00 (a), WXT_1_00 (ab), WXT_4_00 (b)) and MDT (from higher to lower residual percentage and considering the interactions between treatments and week: (WXT_1_00 (a), WXT_4_00 (a), WXT_3_00 (a), WXT_2_00 (b) (significant differences at α = 0.05 were identified by different letters).

Compound	Source of variation	Df	Sum Sq	Mean Sq	F value	P value
PHER	Treatment	3	0.35	0.12	4.02	0.0108
	Week (log-transformed)	1	0.99	0.99	34.54	< 0.0001
	Residuals	67	1.92	0.03		
MDT	Treatment	3	3.11	1.04	6.68	0.0005
	Week (log-transformed)	1	9.76	9.76	62.79	< 0.0001
	Trt * Week (log-transf.)	3	1.55	0.52	3.33	0.025
	Residuals	64	11.50	0.17		

Table S5: Anova table of the linear model investigating the residual quantity of PHER and MDT loaded on NBP for 2023 laboratory trials. Multiple comparisons procedure revealed no difference for PHER. Differences were found for MDT: (from higher to lower residual percentage: NBP_1_FM (a), NBP_4_FM (ab), NBP_2_FM (bc), NBP_3_FM (c)) (significant differences at α = 0.05 were identified by different letters).

Compound	Source of variation	Df	Sum Sq	Mean Sq	F value	P value
PHER	Treatment	3	3.02	1.01	1.59	0.2001
	Week (log-transformed)	1	29.60	29.60	46.79	< 0.0001
	Residuals	67	42.39	0.63		
MDT	Treatment	3	3.88	1.29	4.63	0.0053
	Week (log-transformed)	1	142.48	142.48	510.83	< 0.0001
	Residuals	67	18.6	0.279		

Table S6: Anova table of the linear model investigating the residual quantity of PHER and MDT loaded on BLS for 2024 laboratory trials. Multiple comparisons procedure revealed differences between treatments for PHER (from higher to lower residual percentage and considering the interactions between treatment and week: BLS_SL_FI (a), BLS_SL_FM (ab), BLS_DL_00 (b), BLS_DL_FI (c), BLS_SL_MO (cd), BLS_DL_FM (d), BLS_SL_00 (d), BLS_DL_MO (e)) and MDT (from higher to lower residual percentage and considering the interactions between treatment and week: BLS_SL_FM (a), BLS_SL_FI (ab), BLS_DL_FM (bc), BLS_DL_FI (c), BLS_SL_MO (d), BLS_DL_MO (d), BLS_DL_00 (e), BLS_SL_00 (f)) (significant differences at α = 0.05 were identified by different letters).

Compound	Source of variation	Df	Sum Sq	Mean Sq	F value	P value
PHER	Treatment	7	21.90	3.13	43.24	< 0.0001
	Week (log-transformed)	1	15.71	15.71	217.11	< 0.0001
	Trt. * Week (log-transf.)	7	4.01	0.57	7.90	< 0.0001
	Residuals	188	13.60	0.07		
MDT	Treatment	7	81.30	11.62	92.25	< 0.0001
	Week (log-transformed)	1	148.46	148.46	1179.22	< 0.0001
	Trt. * Week (log-transf.)	7	18.68	2.67	21.19	< 0.0001
	Residuals	194	24.42	0.13		

Table S7: Anova table of the linear model investigating the residual quantity of PHER and MDT loaded on BIP for 2024 laboratory trials. BIP_SL_ML was not evaluated due to a deficiency in the experiment, BIP_DL_MLM because the decrease rate of the two components was already evaluated within the treatments BIP_DL_00 (PHER) and BIP_DL_ML (MDT). Multiple comparisons procedure revealed differences between treatments for PHER (from higher to lower residual percentage and considering the interactions between treatment and week: BIP_SL_FM (a), BIP_SL_00 (a), BIP_DL_00 (ab), BIP_DL_FM (bc), BIP_DL_ML (c)) and MDT (from higher to lower residual percentage and considering the interactions between treatment and week: BIP_DL_00 (a), BIP_SL_FM (b), BIP_SL_00 (b), BIP_DL_FM (b), BIP_DL_ML (b)) (significant differences at $\alpha = 0.05$ were identified by different letters).

Compound	Source of variation	Df	Sum Sq	Mean Sq	F value	P value
PHER	Treatment	4	0.76	0.19	9.57	< 0.0001
	Week (log-transformed)	1	5.57	5.57	281.40	< 0.0001
	Trt. * Week (log-transf.)	4	0.29	0.07	3.63	0.008
	Residuals	98	1.94	0.02		
MDT	Treatment	4	3.47	0.87	6.85	< 0.0001
	Week (log-transformed)	1	48.77	48.77	384.85	< 0.0001
	Trt. * Week (log-transf.)	4	1.47	0.37	2.90	0.026
	Residuals	96	12.16	0.13		

Table S8: Anova table of the linear model investigating the residual quantity of PHER and MDT loaded on BLS used in the 2024 field experiment. Multiple comparisons procedure revealed differences between treatments for PHER (from higher to lower residual percentage: BLS_DL_MO (a), BLS_SL_00 (a), BLS_SL_MO (ab), BLS_DL_00 (ab), BLS_SL_FM (bc), BLS_SL_FI (bc), BLS_DL_FM (c), BLS_DL_FI(c) and MDT (from higher to lower residual percentage: BLS_DL_FI (a), BLS_SL_FM (b), BLS_DL_FM (b), BLS_SL_FI (bc), BLS_DL_MO (cd), BLS_DL_00 (d), BLS_SL_00 (d), BLS_SL_MO (d)) (significant differences at $\alpha = 0.05$ were identified by different letters).

Compound	Source of variation	Df	Sum Sq	Mean Sq	F value	P value
PHER	Treatment	7	24.08	3.44	8.14	0.0003
	Residuals	16	6.76	0.42		
MDT	Treatment	7	7.63	1.09	14.06	< 0.0001
	Residuals	16	1.24	0.08		

Table S9: Anova table of the linear model investigating the residual quantity of PHER and MDT loaded on BIP used in the 2024 field experiment. Multiple comparisons procedure revealed differences between treatments for PHER (from higher to lower residual percentage: BIP_DL_MLM (a), BIP_DL_ML (b), BIP_SL_00 (bc), BIP_SL_ML (bc), BIP_DL_FM (bc), BIP_SL_FM (c), BIP_DL_00 (c)) and MDT (from higher to lower residual percentage: BIP_SL_00 (a), BIP_DL_MLM (ab), BIP_SL_MWL (bc), BIP_SL_FM (cd), BIP_DL_00 (cd), BIP_DL_FM (cd), BIP_DL_ML (d)) (significant differences at α = 0.05 were identified by different letters).

Compound	Source of variation	Df	Sum Sq	Mean Sq	F value	P value
PHER	Treatment	6	25.88	4.31	8.76	0.0004
	Residuals	14	6.89	0.49		
MDT	Treatment	6	2.11	0.35	9.34	0.0003
	Residuals	14	0.53	0.04		

Table S10: Anova table of the linear model investigating trap captures during 2023 field experiment.

Insects	Source of variation	Df	Sum Sq	Mean Sq	F value	P value
Halyomorpha halys adults	Treatment	12	39.31	3.28	7.87	< 0.0001
	Residuals	26	10.82	0.42		
Halyomorpha halys juveniles	Treatment	12	9.38	0.78	0.97	0.50
	Residuals	26	20.87	0.80		
Other stink and leaf-footed bugs	Treatment	12	4.25	0.35	1.43	0.22
	Residuals	26	6.45	0.25		

Table S11: Anova table of the linear model investigating abundance in the surrounding vegetation during 2023 field experiment.

Insects	Source of variation	Df	Sum Sq	Mean Sq	F value	P value
Halyomorpha halys adults	Treatment	12	15.15	1.26	2.78	0.01
	Residuals	26	11.81	0.45		
Halyomorpha halys juveniles	Treatment	12	9.98	0.83	0.74	0.70

	Residuals	26	29.11	1.12		
Other stink and leaf-footed bugs	Treatment	12	11.54	0.96	1.95	0.07
	Residuals	26	12.80	0.49		

Table S12: Anova table of the linear model investigating trap captures during 2024 field experiment.

Insects	Source of variation	Df	Sum Sq	Mean Sq	F value	P value
Halyomorpha halys adults	Treatment	15	13.45	0.90	2.85	0.006
	Residuals	32	10.08	0.31		
Halyomorpha halys juveniles	Treatment	15	13.74	0.92	1.43	0.19
	Residuals	32	20.53	0.64		
Other stink and leaf-footed bugs	Treatment	15	4.55	0.30	1.56	0.14
	Residuals	32	6.22	0.19		

Table S13: Anova table of the linear model investigating abundance in the surrounding vegetation during 2024 field experiment.

Insects	Source of variation	Df	Sum Sq	Mean Sq	F value	P value
Halyomorpha halys adults	Treatment	15	16.36	1.09	1.58	0.14
	Residuals	32	22.06	0.69		
Halyomorpha halys juveniles	Treatment	15	8.89	0.59	0.94	0.53
	Residuals	32	20.12	0.63		
Other stink and leaf-footed bugs	Treatment	15	6.97	0.46	0.92	0.56
	Residuals	32	16.22	0.51		

Table S14: Correlation analysis for 2023 trap captures and abundance in the surrounding vegetation.

Insects	Treatment	Correlation coefficient	P value
Halyomorpha halys adults	BLS_1_00	0.20	0.22
	BLS_2_00	0.30	0.067
	BLS_3_00	0.19	0.25
	BLS_4_00	0.54	0.0004
	NBP_1_FM	0.68	<0.0001
	NBP_2_FM	0.60	<0.0001
	NBP_3_FM	0.56	0.0002
	NBP_4_FM	0.67	<0.0001
	WXT_1_00	0.21	0.21
	WXT_2_00	0.07	0.66
	WXT_3_00	0.44	0.0048
	WXT_4_00	0.36	0.024
	CNT	NA	NA
Halyomorpha halys juveniles	BLS_1_00	0.33	0.039
	BLS_2_00	0.45	0.004
	BLS_3_00	0.56	0.0002
	BLS_4_00	0.52	0.0007
	NBP_1_FM	0.43	0.0069
	NBP_2_FM	0.54	0.0004
	NBP_3_FM	0.42	0.0082
	NBP_4_FM	0.62	<0.0001
	WXT_1_00	-0.13	0.43
	WXT_2_00	0.38	0.018
	WXT_3_00	0.29	0.077
	WXT_4_00	0.39	0.014
	CNT	-0.13	0.43

Table S15: Correlation analysis for 2024 trap captures and abundance in the surrounding vegetation.

Insects	Treatment	Correlation coefficient	P value
Halyomorpha halys adults	BLS_SL_00	0.12	0.43
	BLS_SL_MO	0.33	0.029
	BLS_SL_FI	0.01	0.93
	BLS_SL_FM	0.15	0.33
	BLS_DL_00	-0.13	0.40
	BLS_DL_MO	0.27	0.078
	BLS_DL_FI	0.15	0.32
	BLS_DL_FM	0.13	0.41
	BIP_SL_00	0.11	0.49
	BIP_SL_FM	0.23	0.12
	BIP_SL_ML	-0.02	0.91
	BIP_DL_00	-0.02	0.91
	BIP_DL_FM	0.23	0.13
	BIP_DL_ML	0.05	0.74
	BIP_DL_MLM	0.45	0.0092
	CNT	NA	NA
Halyomorpha halys juveniles	BLS_SL_00	0.52	0.0003
	BLS_SL_MO	0.27	0.068
	BLS_SL_FI	0.42	0.0042
	BLS_SL_FM	0.16	0.31
	BLS_DL_00	0.28	0.063
	BLS_DL_MO	0.38	0.0098
	BLS_DL_FI	0.28	0.061
	BLS_DL_FM	0.45	0.0017
	BIP_SL_00	0.28	0.058
	BIP_SL_FM	0.37	0.013
	BIP_SL_ML	0.36	0.016
	BIP_DL_00	0.20	0.18
	BIP_DL_FM	0.24	0.11
	BIP_DL_ML	0.48	0.0008

	BIP_DL_MLM	0.61	0.0002
	CNT	NA	NA
