# Supplementary material for: Evaluation of Lure and Dispenser Combinations for Halyomorpha halys (Hemiptera: Pentatomidae) Trapping
Source: Insects. 2025 Mar 25;16(4):341. doi: 10.3390/insects16040341 (PMC12028315; doi:10.3390/insects16040341)
Supplement: Supplementary file 1 [file insects-16-00341-s001.zip › insects-3365970-supplementary_Figures_R1.pdf]

Evaluation of Lure and Dispenser Combinations for *Halyomorpha halys* (Hemiptera: Pentatomidae) Trapping

Vito Antonio Giannuzzi [1,†], Valeria Rossi [1,†], Rihem Moujahed [2], Adriana Poccia [1], Florinda D'Archivio [1], Tiziano Rossi Magi [1], Elena Chierici [1], Luca Casoli [3], Gabriele Rondoni [1,*] and Eric Conti [1]

1 Department of Agricultural, Food and Environmental Sciences, University of Perugia, Borgo XX Giugno, 74, 06121 Perugia, Italy; vitoantonio.giannuzzi@dottorandi.unipg.it (V.A.G.); valeria.rossi@unipg.it (V.R.); adriana.poccia@dottorandi.unipg.it (A.P.); florinda.darchivio@gmail.com (F.D.); tizianorossimagi97@gmail.com (T.R.M.); elenachierici9@gmail.com (E.C.); eric.conti@unipg.it (E.C.)
2 Russell IPM Ltd., Deeside CH5 2NU, UK; rihem@russellipm.com
3 Consorzio Fitosanitario di Reggio Emilia, 42124 Reggio Emilia, Italy; luca.casoli@regione.emilia-romagna.it
* Correspondence: gabriele.rondoni@unipg.it
† These authors contributed equally to this work.

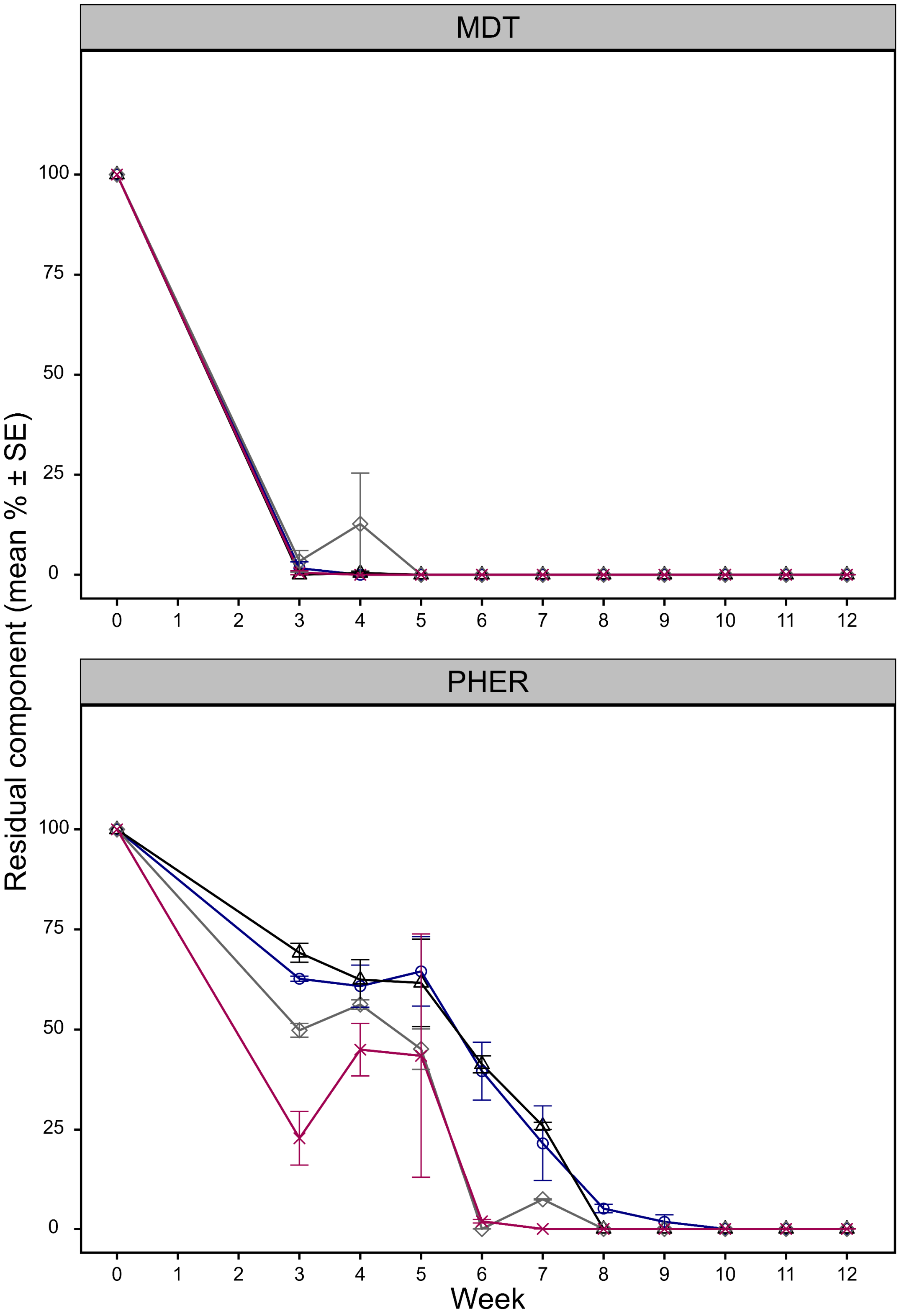

Figure S1. Residual quantity of MDT and PHER in BLS dispenser during 2023 laboratory trial. Pink = BLS_1_00, black = BLS_2_00, navy-blue = BLS_3_00, gray = BLS_4_00. For details on the compound combinations please refer to Table 1 of the main manuscript.

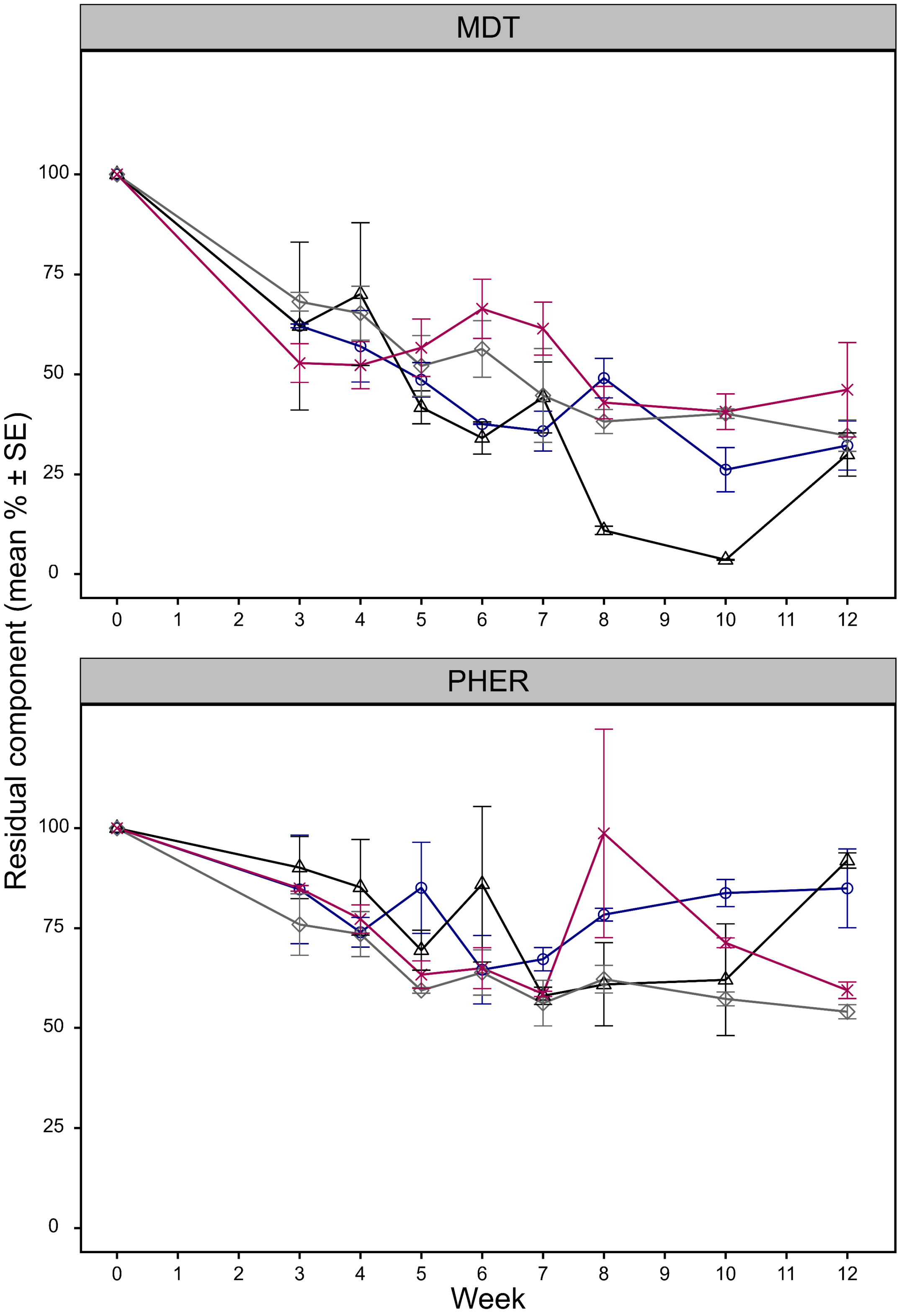

Figure S2. Residual quantity of MDT and PHER in WXT dispenser during 2023 laboratory trial. Pink = WXT_1_00, black = WXT_2_00, navy-blue = WXT_3_00, gray = WXT_4_00. For details on the compound combinations please refer to Table 1 of the main manuscript.

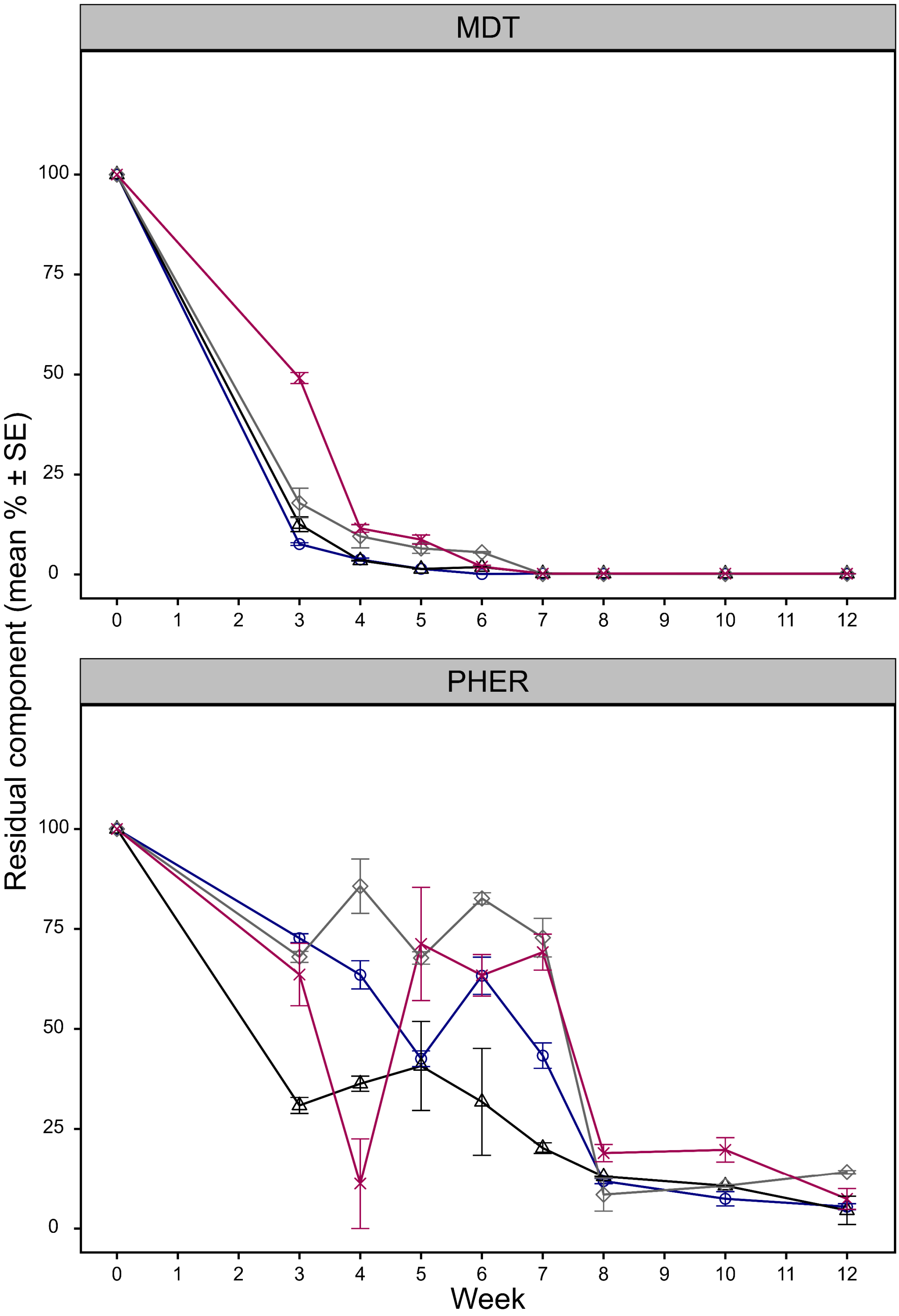

Figure S3. Residual quantity of MDT and PHER in NBP dispensers during 2023 laboratory trial. Pink = NBP_1_FM, black = NBP_2_FM, navy-blue = NBP_3_FM, gray = NBP_4_FM. For details on the compound combinations please refer to Table 1 of the main manuscript.

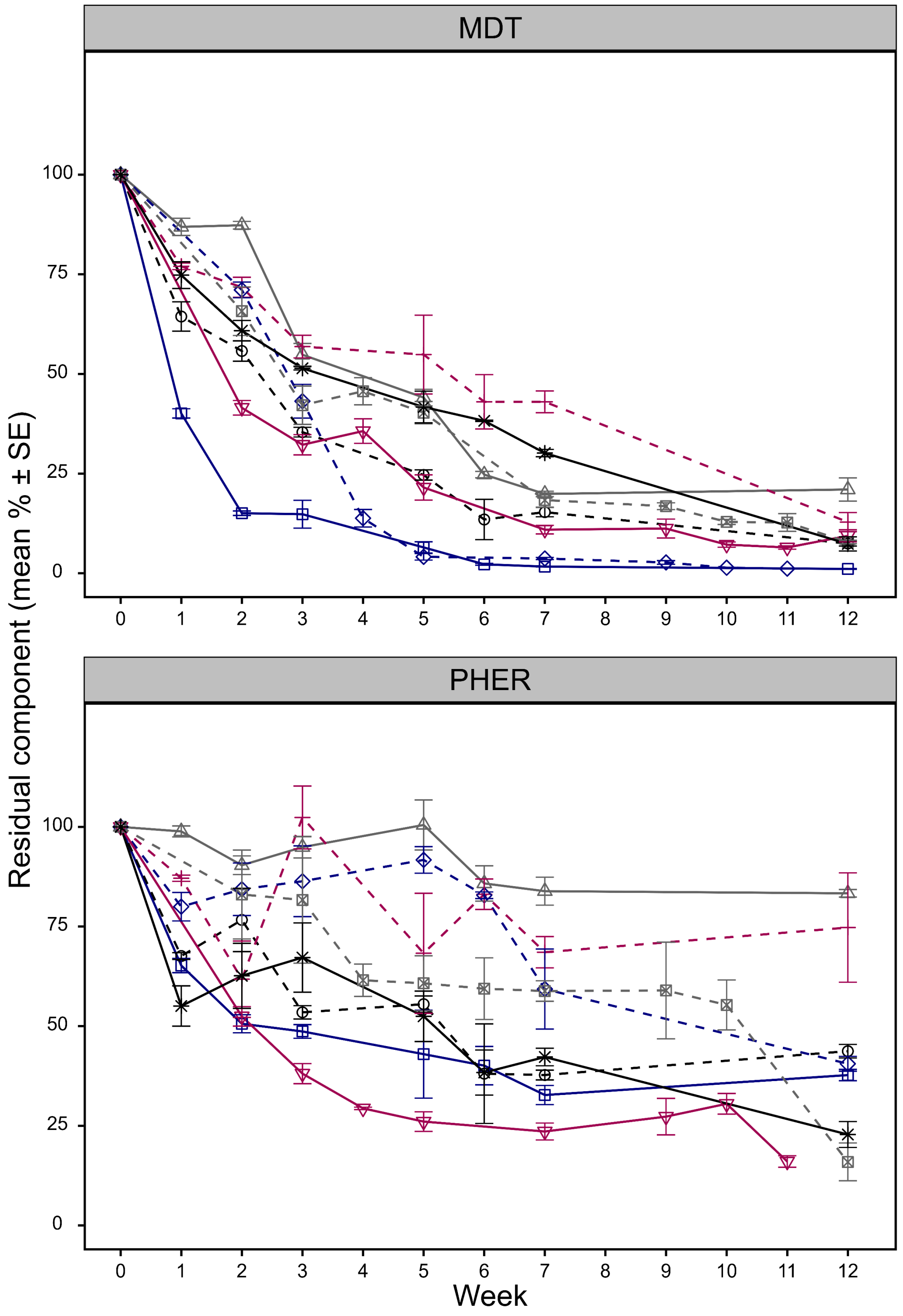

Figure S4. Residual quantity of MDT and PHER in BLS dispensers during 2024 laboratory trial. Navy-blue = BLS_SL_00, solid line; BLS_DL_00, dashed line. Black = BLS_DL_FM, solid line; BLS_SL_MO, dashed line. Gray = BLS_SL_FI, solid line; BLS_DL_FI, dashed line. Pink = BLS_DL_MO solid line; BLS_SL_FM, dashed line. For details on the treatments please refer to Table 1 of the main manuscript.

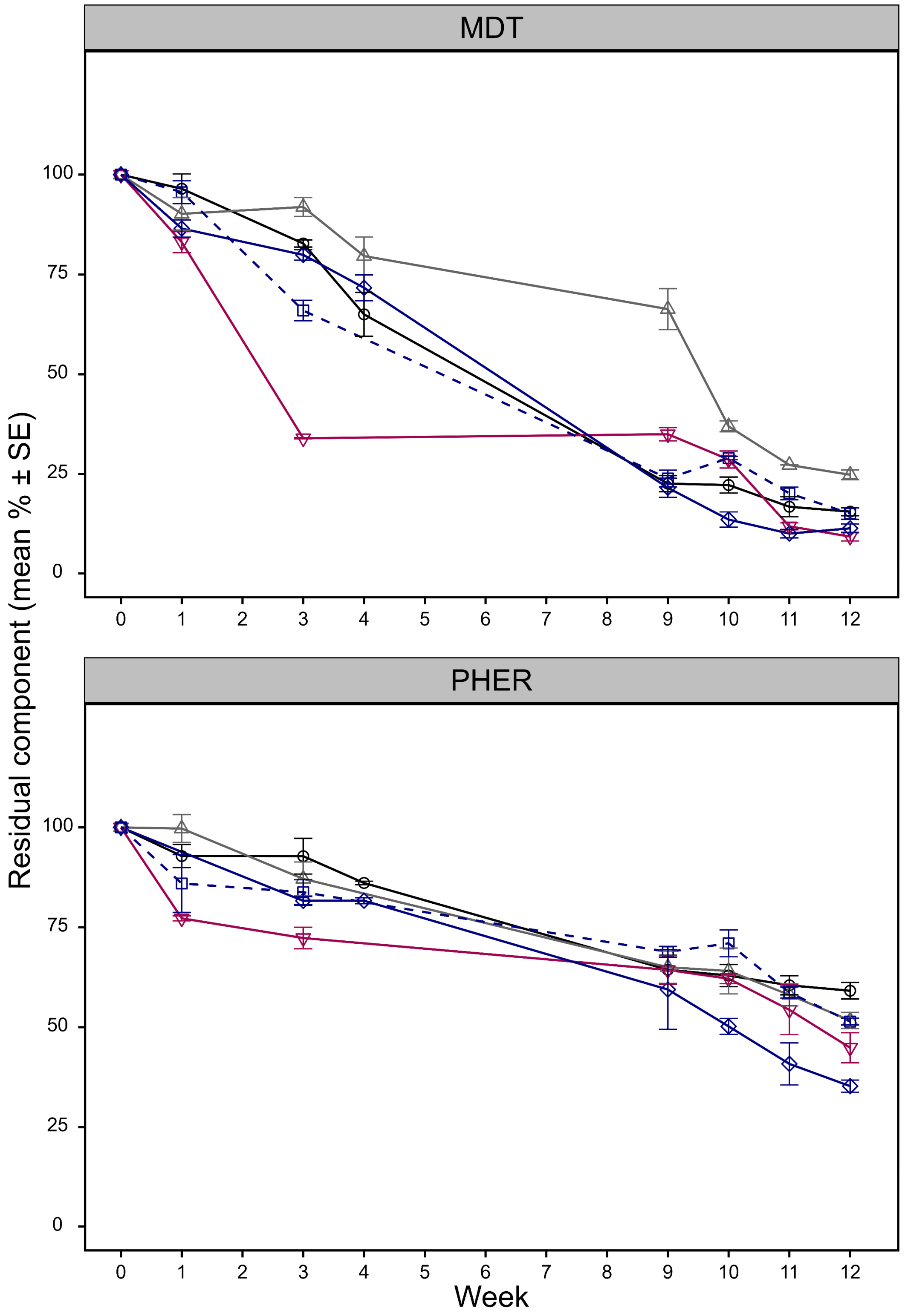

Figure S5. Residual quantity of MDT and PHER in BIP dispensers during 2024 laboratory trial. Navy-blue = BIP_DL_ML, solid line; BIP_SL_00, dashed line. Black = BIP_SL_FM. Gray = BIP_DL_00. Pink = BIP_DL_FM. For details on the compound combinations please refer to Table 1 of the main manuscript.

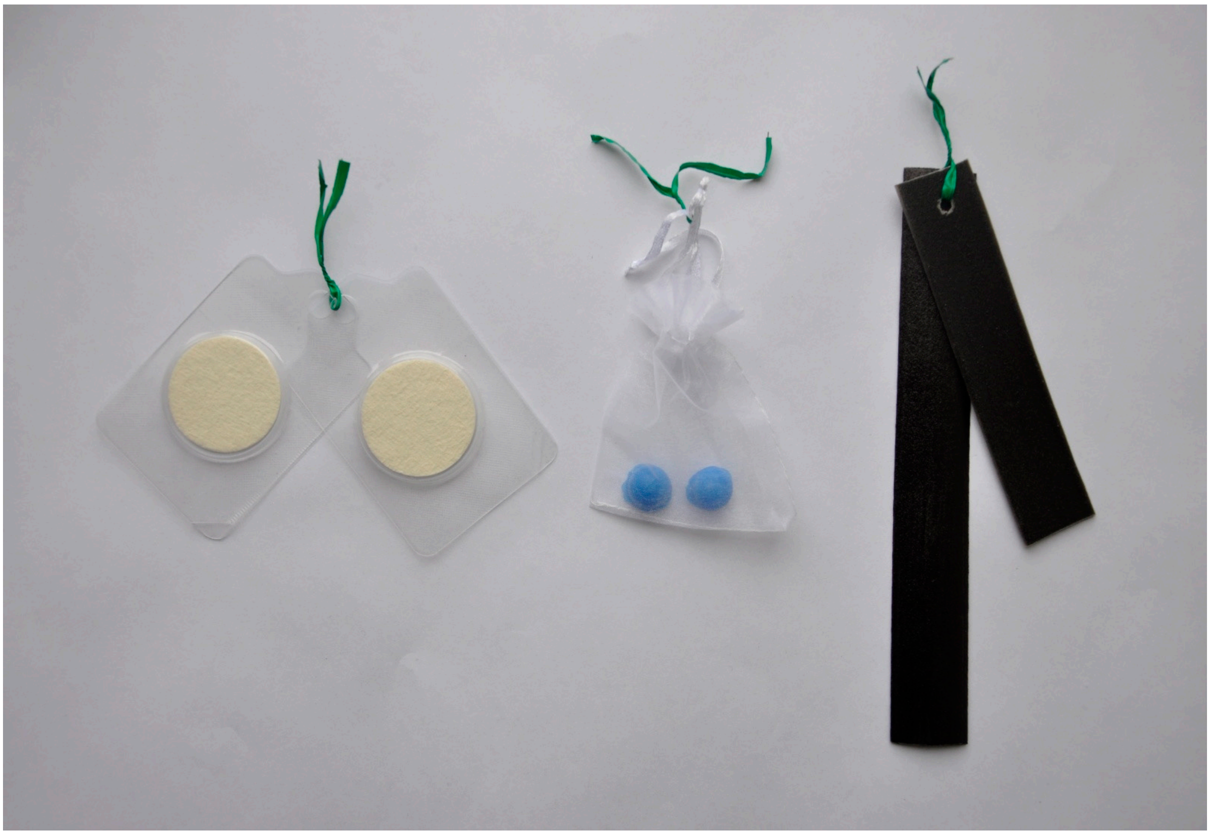

Figure S6. Types of dispensers used in the 2023 field trial. From left to right: Blister Pack (BLS), Wax Tablets (WXT), and Non-Biodegradable Polymer (NBP).

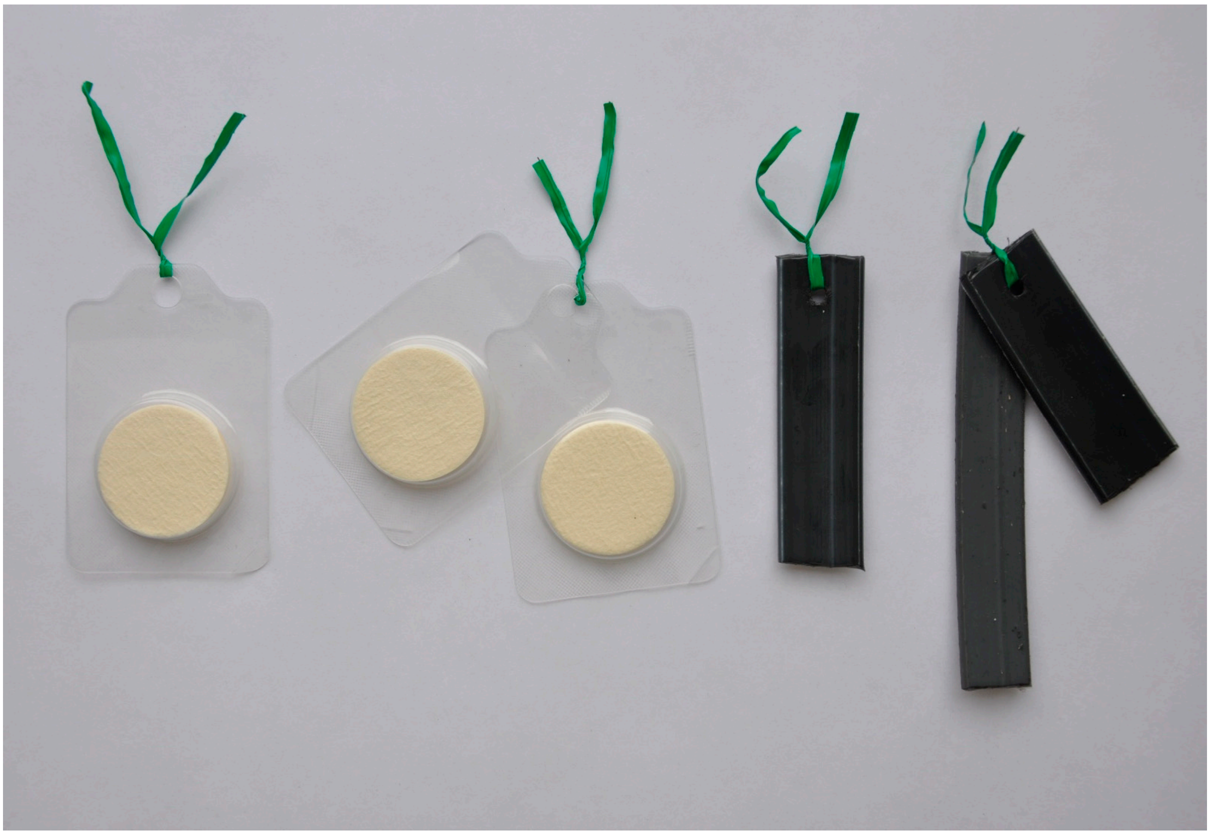

Figure S7. Types of dispensers used in the 2024 field trial. From left to right: Blister Pack Single Lure (BLS_SL), Blister Pack Dual Lure (BLS_DL), Biodegradable Polymer Single Lure (BIP_SL), and Biodegradable Polymer Dual Lure (BIP_DL).
